# Wild Skylarks Seasonally Modulate Energy Budgets but Maintain Energetically Costly Inflammatory Immune Responses throughout the Annual Cycle

**DOI:** 10.1371/journal.pone.0036358

**Published:** 2012-05-03

**Authors:** Arne Hegemann, Kevin D. Matson, Maaike A. Versteegh, B. Irene Tieleman

**Affiliations:** Animal Ecology Group, Centre for Ecological and Evolutionary Studies, University of Groningen, Groningen, The Netherlands; Johns Hopkins University School of Medicine, United States of America

## Abstract

A central hypothesis of ecological immunology is that immune defences are traded off against competing physiological and behavioural processes. During energetically demanding periods, birds are predicted to switch from expensive inflammatory responses to less costly immune responses. Acute phase responses (APRs) are a particularly costly form of immune defence, and, hence, seasonal modulations in APRs are expected. Yet, hypotheses about APR modulation remain untested in free-living organisms throughout a complete annual cycle. We studied seasonal modulations in the APRs and in the energy budgets of skylarks *Alauda arvensis*, a partial migrant bird from temperate zones that experiences substantial ecological changes during its annual cycle. We characterized throughout the annual cycle changes in their energy budgets by measuring basal metabolic rate (BMR) and body mass. We quantified APRs by measuring the effects of a lipopolysaccharide injection on metabolic rate, body mass, body temperature, and concentrations of glucose and ketone. Body mass and BMR were lowest during breeding, highest during winter and intermediate during spring migration, moult and autumn migration. Despite this variation in energy budgets, the magnitude of the APR, as measured by all variables, was similar in all annual cycle stages. Thus, while we find evidence that some annual cycle stages are relatively more energetically constrained, we find no support for the hypothesis that during these annual cycle stages birds compromise an immune defence that is itself energetically costly. We suggest that the ability to mount an APR may be so essential to survival in every annual cycle stage that skylarks do not trade off this costly form of defence with other annual cycle demands.

## Introduction

Seasonal variation in immune function has been found in a variety of vertebrate taxa and has been attributed to seasonally changing annual cycle demands, resource availability and exposure to pathogens and parasites [1], [2]. Because production, maintenance and use of the immune system require energy [3], [4], a central hypothesis states that immune defences, particularly those components that have high costs, are traded off against other competing physiological and behavioural processes [5]–[7]. Such trade-offs putatively explain findings of reduced immune responses in relation to reproduction [3], [8], [9], during migration [10], [11] or during winter [12]. Furthermore the outcome of such trade-offs is affected by the evolutionary pressures exerted by pathogens and parasites [13], which may also change throughout the year. In addition to trade-offs between the immune system and other physiological systems, trade-offs within the immune system may also occur. For example, organisms may shift from more to less costly defences during times of high energy demand or low resource availability [2], [14]. More specifically, Lee [15] hypothesizes a switch from costly inflammatory responses to highly specific but less costly antibody responses.

The acute phase response (APR), an innate response that is initiated minutes after detecting an inflammatory agent, is an early defence against threats that have already breached physical barriers like the skin. APRs involve an array of physiological and behavioural changes, including fever and anorexia [16], and these responses incur costs from metabolic upregulation and tissue degradation [5], [17], [18]. In birds, potential proximate mechanisms underlying seasonal changes in APRs are hypothesized to include hormonal suppression and seasonal differences in energy stores [16], [19]. Thus far, studies of seasonal modulations in APRs consider only two annual cycle stages [19] and have been done on either captive birds or wild birds that have been in captivity for at least several weeks [20], [21]. While offering some insight, the conclusions of these studies are limited by the lack of a complete year-round perspective on immune function and by lack of simultaneous measurements of the energy budget.

To identify which annual cycle stages are energetically demanding, ecologists quantify indices of energy metabolism [22], [23]. Basal metabolic rate (BMR) is the most standardized measure [24], and BMR relates to many other ecologically-important variables including activity level [25], [26], food availability and diet [27], [28], organ sizes and body composition [29]–[31], and daily energy expenditure [29], [32], [33]. These relationships make BMR an interesting trait for ecological studies of seasonal variation. Metabolism represents only part of the energetic balance, and energetic challenges can also result from limitations on resource availability. Thus, data on body mass and biochemical markers can provide critical information about whether birds obtain nutrients from available food or from body reserves. Two such biochemical markers are glucose and ketone. Glucose is one of the main sources for energy production in birds [34] and the primary carbohydrate absorbed by the avian intestine [35]. Ketone concentrations reflect lipid catabolism during fasting [36]–[38].

Most temperate zone birds experience substantial changes in their ecology over the course of a year. Energy and time budgets change in association with seasonal activities like migration and reproduction and with variable environmental conditions like temperature and precipitation. The skylark, *Alauda arvensis*, is a typical temperate zone passerine and a partially migratory species, with migration dependent on breeding location [39], [40]. During an annual cycle, skylarks go through five distinct annual cycle stages: spring migration, breeding, moult, autumn migration, winter. With transitioning stages, skylarks face changes in environmental conditions, social structure (pairs during breeding, flocks outside the breeding season), and diet (predominantly insectivorous during summer, predominantly granivorous during winter) [41].

To understand how seasonal changes in energy budgets relate to seasonal changes in immune function, simultaneous measurements of both are needed throughout the year in the same species. So far, only components of this design have been investigated. For example, studies of the energy budget of skylarks are restricted to the breeding season. These studies show that the field metabolic rate of breeding skylarks is 11% below allometric predictions and that the ratio between field metabolic rate and BMR is 1.7 [42], [43], which is substantially below the optimal working capacity of four-times-BMR for birds tending broods proposed by Drent and Daan [44]. Measurements during other annual cycle stages are required to determine if metabolism levels during breeding are comparatively low. Studies of baseline immune function in skylarks and other birds show differences in indices among different annual cycle stages, but these studies have been measured on un-challenged birds and in isolation of energetic measurements [41], [45]–[47]. Nevertheless, these studies suggest that patterns of immune function are linked to changing environmental conditions [41], [47]. To our best knowledge no studies linking modulations in the energy budgets over a complete annual cycle to modulations in energetically-costly immune responses have been carried out in any free-living vertebrate.

We studied seasonal modulations of energy budgets and APRs in wild skylarks across the species' entire annual cycle. To characterize changes in the energy budget, we measured body mass and basal metabolic rate. To quantify the energetic and nutritional costs of activating the APR, we measured the effects of a lipopolysaccharide injection on metabolic rate, body mass, body temperature, and concentrations of glucose and ketone. Based on the hypothesis that birds should compromise expensive immune responses during energetically-demanding times of the annual cycle, we expected seasonal modulations in the magnitude of the APR to occur in relation to changes in the energy budget. Since males and females are hypothesized to differently allocate resources to their immune system [2], [14], we also investigated if the sexes mount APRs of different magnitudes.

## Materials and Methods

### Ethics Statement

The study described here was specifically approved by the Institutional Animal Care and Use Committee of the University of Groningen under license DEC5219B. The populations study in the Aekingerzand was done under licence D4743A and DEC5219B of the same committee and all their guidelines and conditions were strictly followed.

### Birds and field capture

We caught skylarks during five annual cycle stages in the northern Netherlands in 2008 and 2009 focusing on our study population at the Aekingerzand (N 52°55′; E 6°18′). Skylarks in our study population are partial migrants. Some birds migrate; others winter locally and are accompanied by birds that breed further north and east [48]. During breeding (15 Jun–7 Jul 2008), we caught birds (9 m, 6 f) that were feeding nestlings with mist-nests or traps on the nest from our study population at the Aekingerzand. Birds were caught in the afternoon and released early next morning to minimize the time adults were absent from their nest. From three nests we took both parents but at different times to help ensure continued food provisioning for the nestlings. During molt (3 Aug–22 Sep 2008), we caught birds (12 m, 7 f) from the same population by flushing birds into nets during the night. During winter (9 Dec 2008–15 Jan 2009) birds from the study population use agricultural fields that surround the core study area [48]. We caught birds (14 m, 3 f) on these fields by flushing them into nets at night. During migration in spring (14 Mar–24 Mar 2008 and 25 Feb–1 Mar 2009; 12 m, 12 f) and autumn (9 Oct–2 Nov 2008; 17 m, 9 f), we caught actively migrating birds with clap-nets during periods of visible diurnal skylark migration at a location about 15 km southeast of the Aekingerzand. We are confident about the migratory status: when tape-lured, migrants interrupt their migratory flight, but local birds that are not currently migrating do not respond.

Upon capture, we punctured the brachial vein with a sterile needle and collected blood samples into heparinised capillary tubes before taking structural measurements. Birds were sexed biometrically and some doubtful cases were sexed molecularly [49]. All individuals were fully grown. Because skylarks undergo a complete post-nuptial moult in August–September, age classes could not be distinguished. Since skylarks breed in their first year (Hegemann unpublished data) and both young and adult birds are known to migrate [48], [50], we have no indications that an age bias between stages exists and could influence the interpretation of the results.

### Experimental protocol and respirometry setup

After capture, we brought birds into captivity and, assigned each one to either the experimental or control group. We balanced these groups for sex. Because birds were caught at different times of the day and because the respirometry setup could measure a maximum of three individuals per night, time in captivity varied. Of the 101 skylarks, 76 spent <24 h in captivity before the experiment started (median: 17:40 h; minimum: <2 h (n = 2); maximum: 69 h (n = 2)). We housed up to three birds per cage (30×40×60 cm) during all annual cycle stages except the breeding season, when skylarks were territorial and housed individually. Prior to initiation of the experimental protocol, birds had access to water, mealworms and seeds *ad libitum*.

At the start of the experimental protocol at 4.30 p.m, food and water were withdrawn and birds were isolated in a dark box for 1 h. At 5:30 p.m., we collected baseline pre-metabolic body temperatures and masses of all birds, and we injected the experimental birds with LPS. We inserted a thermocouple about 1 cm into the cloacae and recorded the temperature (OMEGA ATT thermometer) to the nearest 0.1°C once the temperature was stable for 10 sec., and we measured body mass to the nearest 0.1 g. Experimental birds were injected intra-abdominally with 2.5 mg LPS in 10 mL PBS per kg body mass.

The LPS dose was based on results of a pilot study in skylarks and rooted in published data. In Japanese quail *Coturnix coturnix japonica*, a dose of 0.5 mg LPS per kg body mass does not lead to a significant response, and doses higher than 2.5 mg/kg do not lead to additional increases in the response magnitude [51]. Thus, we first tested the effects of 1.0 mg/kg (n = 3) and 2.5 mg/kg (n = 3) in Skylarks. These birds responded more strongly to the higher dose in terms of average mass-corrected metabolic rate (2.75 vs. 2.52 ml O2/hr/g) and body temperature 13 hours post-injection (40.8 vs. 40.0°C). No birds died following injection with either dose of LPS. Combined, these results suggested to us that the higher dose (2.5 mg/kg) was appropriate for our current study and that it led to a greater response than would be possible from the vehicle (PBS) alone.

LPS injections mimic bacterial infection without resulting in infection. Control birds remained un-injected because puncturing the skin and other tissues and injecting a vehicle (e.g. PBS only) can also result in inflammation (K. Klasing and B. Helm, personal communications). Consequently, the experimental responses must be viewed as a result of both the LPS and the injection procedure. This combination of effectors does not pose interpretational problems for our study since our central interest is inflammation versus the absence of inflammation and not the effects of LPS per se.

Immediately after measuring body temperature and body mass from all birds and injecting the experimental birds, birds were sealed individually in airtight metabolic chambers. The metabolic chambers sat inside a larger environmental chamber that was set to 30°C, which is within the thermoneutral zone of skylarks [42]. The first 1.5 hours in the chamber served as acclimation and equilibration period. We recorded O_2_-consumption and CO_2_-production from 7:00 p.m. to 6:30 a.m. the following morning using standard flow-through respirometry [52]. Briefly, compressed ambient air was dried and pumped through calibrated mass-flow controllers (model 5850S; Brooks Instrument, PA, USA) at 40 L h^−1^ and through the metabolic chambers. After leaving the chambers, the air passed through silica gel filters to remove the moisture added by the birds (e.g. through respiration). Then, the percentages of O_2_ in the air were measured with gas analyzers (O_2_: Servomex Xentra 4100, Crowborough, UK). A reference stream of dried air that bypassed the metabolic chambers was analysed at least once every two hours.

We calculated O_2_ consumption (mL h^−1^ g^−1^) using equations adapted from Hill [53]. Nightly metabolic rate was calculated as the average O_2_ consumption per bird between 7:00 pm and 6:30 am. BMR was calculated as the lowest average O_2_ consumption during any 12 min interval during the night.

At 6:30 a.m. (14 h after the start of the experimental protocol and after 11.5 h of metabolic measurements) we took birds out of the chambers, immediately measured body temperature, collected a blood sample and re-measured body mass. All data and samples were collected <10 min after opening the metabolic chamber. We used ∼15 uL of fresh blood to measure glucose and ketone concentrations with a handheld diagnostic device (CardioChek PA Analyzer 1708 with glucose test strips 1713 and ketone test strips 1718; Polymer Technology Systems, IN, USA). Ketone concentrations were not measured during autumn migration. Upon completion of the entire protocol, birds were released at the site of capture.

### Effects of duration in captivity

We conducted a pilot study to compare effects of short term and longer term captivity. Three skylarks were held in captivity for 8 hours (short); four other skylarks were acclimated to captivity over 55 days (long). Following these captivity periods, we subjected the birds to the protocol of this study. We found no difference between these two groups in their responses to a challenge with 1.0 mg LPS/kg body mass (e.g., mass loss: short = 9.0±1.1% (SD), long = 9.9±2.2%, t(5) = 0.6, P = 0.6; O_2_ consumption: short = 3.3±0.5 mL/hr/g, long = 3.5±0.8, t(5) = 0.4, P = 0.7). While the stress of short term captivity did not appear to affect these metabolic parameters of APRs, captive birds generally differ from their wild counterparts in many other ways (e.g., nutrition, activity). As a result, we favoured studying birds that were in captivity for as short a period as possible.

We also explored if stress from short-term captivity affected birds differently in different annual cycle stages. We used heterophil/lymphocyte ratios and concentrations of heat shock protein 70 as indicators for stress [54]–[56], and we found no differences in the effects of captivity among annual cycle stages.

### Statistics

We compared experimental and control groups for each response variable using linear models analysed with the program R version 2.9.2 [57]. We included sex, annual cycle stage, treatment and all possible interactions as explanatory variables. We always started with the full model and simplified it using backwards elimination based on log likelihood ratio test with P<0.05 as selection criterion (“drop1" in R) until reaching the minimal adequate model. Assumptions of all models were checked on the residuals of the final model. Graphs were made using the package “gplots" [58]. Sample sizes differ slightly among response variables due to technical problems (e.g. thermometer failure).

Experimental and control groups did not differ significantly in body mass (χ^2^_1,99_ = 1.95, P = 0.17) or glucose concentration (χ^2^_1,87_ = 0.03, P = 0.86) when measured in the field just after catching and thus well in advance of the LPS injection. We also found no significant pre-metabolic differences in body mass (χ^2^_1,99_ = 1.42, P = 0.24) and body temperature (χ^2^_1,99_ = 0.65, P = 0.42) between the two treatment groups immediately prior to the LPS injection.

## Results

### Seasonal modulation in body mass and basal metabolic rate

**Figure 1:**
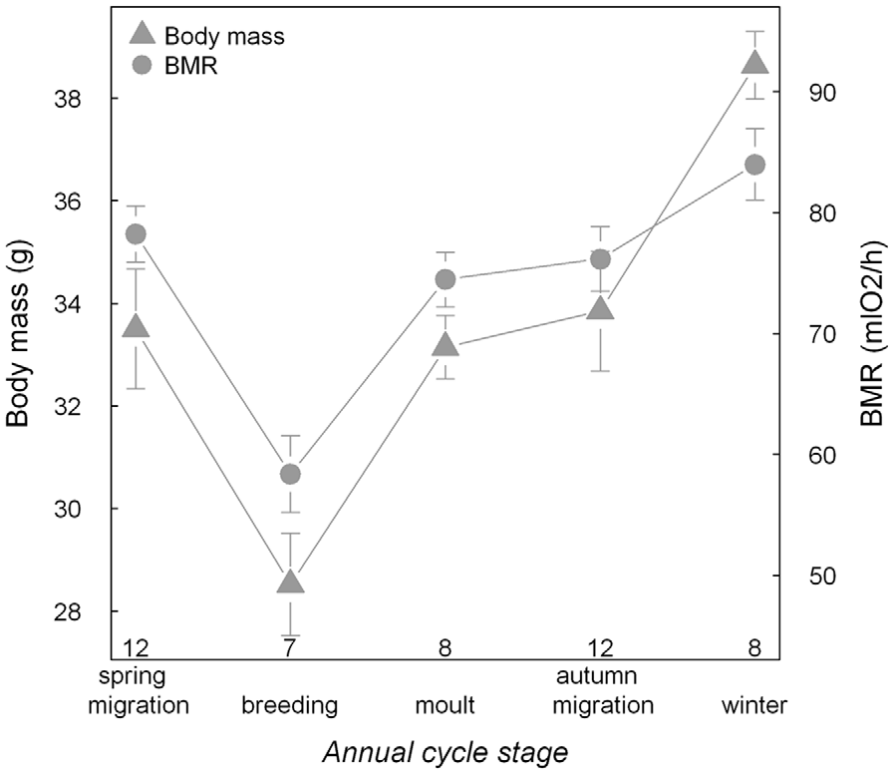
Body mass and mass-specific basal metabolic rate (BMR) of control skylarks throughout the annual cycle. Body mass measurements were taken in the mornings upon completion of the metabolic measurements. Numbers represent samples sizes.

Body mass and BMR of skylarks varied among annual cycle stages (χ^2^_4,46_ = 34.66, P<0.001; χ^2^_4,46_ = 31.46, P<0.001; Figure 1), suggesting a seasonal modulation of the energy budget. Both body mass and BMR were lowest during the breeding season and highest during winter (Figure 1). During spring migration, moult and autumn migration values of body mass and BMR were comparable and intermediate.

### Seasonal modulation of the acute phase response

**Figure 2:**
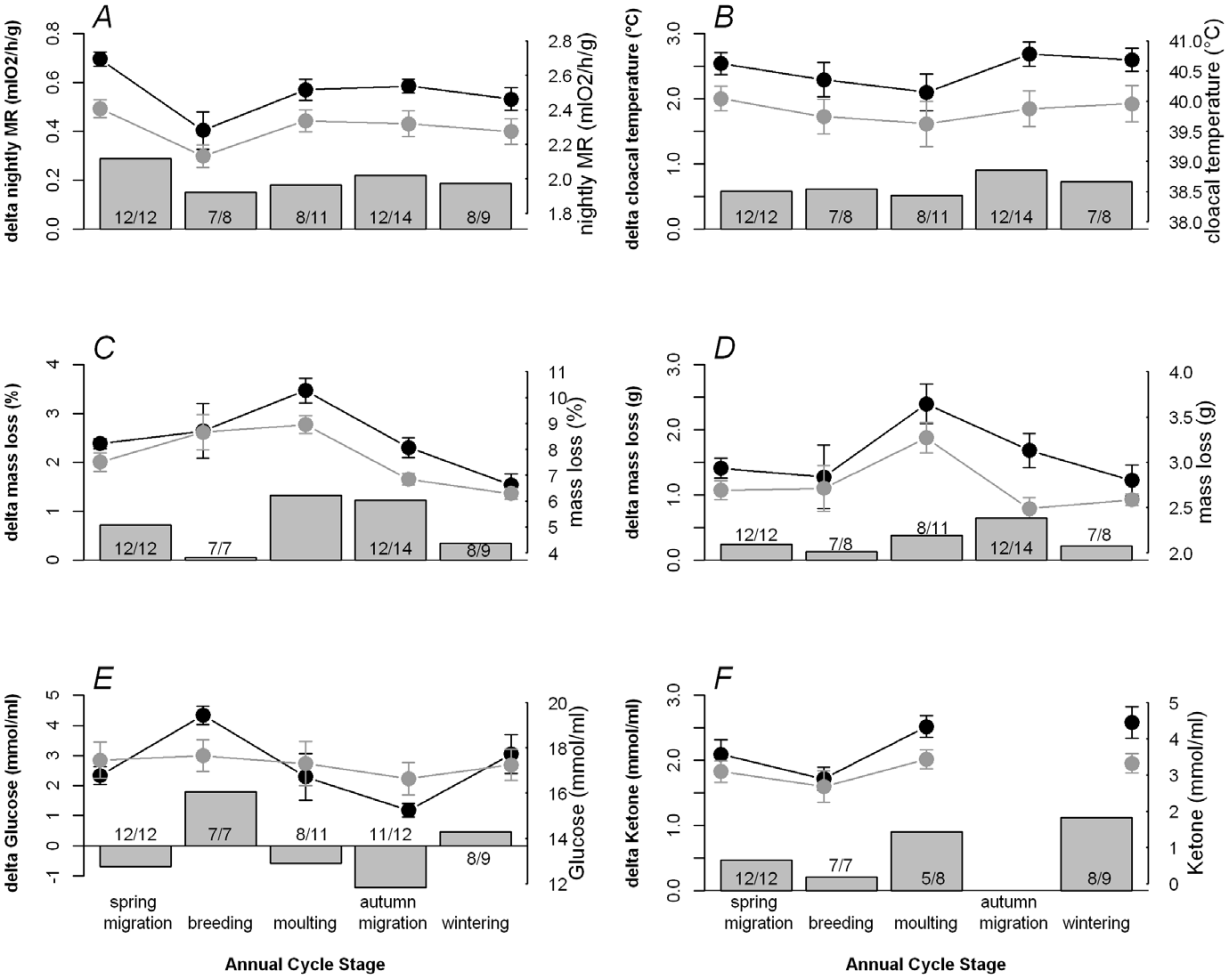
Effect of LPS injection on a) mass-specific nightly metabolic rate, b) body temperature, c) relative mass loss during the night, d) absolute mass loss during the night, e) glucose concentration and f) ketone concentration of skylarks after 13 hours. Experimental birds were injected with LPS; control birds were un-injected. Black symbols represent experimental birds (LPS-injected), grey symbols control birds (un-injected). Bars represent the difference between these two groups. Means and standard errors are shown; numbers in bars represent sample sizes per group (control/experimental). The graphs show raw data without correcting for sex effects. There was never a significant treatment* annual cycle stage interaction (all P>0.084). See Table 1 for statistics.

The experimentally-induced APR led to increases in metabolic rate, body temperature, mass loss and ketone concentrations, but we found no evidence for different effects of LPS in different annual cycle stages (Table 1, Figure 2A–F; body masses relevant for interpretation of metabolic measures are provided in Table 2). For every variable, we removed the non-significant interaction term (treatment*annual cycle stage) before testing the main effects of LPS injection and annual cycle stage. Both were significant (Table 1).

**Table 1:** Statistics and coefficients of the linear models of measures of metabolism, body mass and temperature in skylarks.

Trait	Annual cycle stage			Sex				Treatment				Treatment×Annual cycle stage		
	df	Chi^2^/F	p	df	Chi^2^/F	p	β†	df	Chi^2^/F	p	β‡	df	Chi^2^/F	p
Nightly metabolic rate°	100,4	27.56	<0.001	100,1	13.36	<0.001	0.144	100,1	28.04	<0.001	0.205	100,1	2.54	0.639
Basal metabolic rate°	100,4	37.41	<0.001	100,1	6.17	0.013	0.087	100,1	25.66	<0.001	0.176	100,1	3.35	0.502
Body mass loss, grams*	100,4	25.77	<0.001	100,1	15.16	<0.001	−0.441	100,1	12.16	<0.001	0.371	100,1	3.66	0.454
Body mass loss, %*	100,4	45.48	<0.001	100,1	0.02	0.884		100,1	8.18	0.004	0.796	100,1	3.32	0.506
Body temperature	98.4	5.72	0.221	98,1	1.80	0.180		98,1	15.49	<0.001	0.668	98,1	1.23	0.873
[Glucose]	96,4	12.10	0.017	96,1	0.47	0.495		96,1	0.31	0.575		96,1	5.69	0.223
[Ketone]	67,3	14.39	0.002	67,3	7.48	0.006	0.717	67,3	5.69	0.017	0.590	67,3	2.26	0.521

† Reference category is ‘male’;

‡ Reference category is ‘control’;

° mass-specific;

* Calculated over the 13 h experimental period.

Experimental birds were injected with LPS; control birds were un-injected. Results are from linear models after removing all non significant terms (P>0.05). All tests are based on chi-square statistics.

**Table 2:** Body mass of skylarks per annual cycle stage after 13 h of nightly metabolic measurements.

	Body mass (in g ± s.e.)	
	Control	Experimental
Spring migration	33.5±1.17	32.7±0.80
Breeding	28.5±1.00	29.9±1.13
Moult	33.1±0.62	31.7±0.87
Autumn migration	33.9±1.16	35.5±0.97
Winter	38.6±0.67	39.7±1.26

Experimental birds were injected with LPS; control birds were un-injected.

LPS injection caused a significant increase in mass-specific nightly metabolic rate, mass-specific BMR, body mass loss, body temperature and ketone concentrations (Table 1). On average, the LPS injection increased mass-specific nightly metabolic rate by 9.06% (Figure 2A), body temperature by 0.7°C (Figure 2B), increased mass loss by 0.6 g or 13.6% (Figure 2C, D). Glucose concentrations did not significantly change in response to LPS injection (Table 1, Figure 2E).

Annual cycle stages differed in mass-specific nightly metabolic rate, body mass loss, glucose and ketone concentrations (Table 1). Moreover, the shape of the seasonal patterns differed among these variables (Figure 2). Mass-specific nightly metabolic rate (Figure 2A) followed the patterns of BMR (Figure 1) with a dip during breeding. Nightly mass loss in percentage or absolute terms was highest during moult and lowest during winter (Figure 2C, D). Glucose concentration was highest during breeding and lowest during autumn migration (Figure 2E); ketone concentration was lowest during breeding and high during moult and winter (Figure 2F).

### Sex differences

Males and females differed significantly in mass-specific nightly metabolic rate, nightly body mass loss in grams and ketone concentrations (Table 1). We found no differences between the sexes among annual cycle stages (sex by annual cycle stage always F<5.56 and P>0.23) or in their response to the LPS injection (sex by LPS injection always F<1.32 and P>0.25). Compared with males, females had a 7.2% higher mass-specific nightly metabolic rate and a 18.9% higher ketone concentration. Males lost more grams of body mass during the night than females (males 3.06 g, female 2.68 g), but this effect was proportional to the difference in body mass between the sexes, and disappeared when relative mass loss was considered (males 7.9%, female 8.1%).

## Discussion

We found that the acute phase responses of skylark was consistent among five annual cycle stages (spring migration, breeding, moult, autumn migration, winter). This constancy of inflammatory responses contrasted sharply with the observed seasonal variability in the skylark energy budget, which was reflected by changes among annual cycle stages in terms of energy metabolism, body mass and concentrations of glucose and ketone. Thus, while we find evidence that some annual cycle stages are relatively more energetically constrained, we find no support for the hypothesis that during these annual cycle stages birds compromise an immune defence that is itself energetically costly.

### Testing the trade-off hypothesis

Our results clearly demonstrate that the inflammation caused by an LPS injection was energetically costly for skylarks, but we have no evidence for seasonal modulation of the inflammatory response in this species. A lack of seasonal modulation contradicts current hypotheses relating compromised inflammatory responses with other life-history demands [2], [16], [59]. Consistent APRs throughout the annual cycle might signal that this innate defence is simply too important to be compromised. Maintenance of this response, however, does not rule out possible trade-offs with other physiological and behavioural processes. For example, some birds change their territorial behaviour in response to an inflammatory challenge [19], [20]. If inflammatory responses are indeed linked to broader physiological functioning via a resource budget and the responses are seasonally consistent as our data suggest, then birds undergoing an APR might be forced to reduce the resources they spend on other traits, for example by delaying migration [60], reducing parental effort [3] or postponing moult [61]. These types of adjustments support the life-history trade-off hypothesis [16] in the sense that, instead of the immune system being compromised, other annual cycle events are suppressed, which in turn may negatively impact individual fitness.

Skylarks in our study population are partial migrants, with some birds migrating and others wintering locally [48]. We chose to focus our study on the year-round inflammatory responses of skylarks in the breeding location, reflecting that part of our study population that winters locally. Because birds from northern and eastern populations join the local skylark population outside breeding and moult, we potentially caught a mixture of birds from different populations during winter and migration. We explored if this led to larger coefficients of variation (CV) for the various response variables, but found no difference in the CV during winter and migration in comparison with breeding and moult. This is in agreement with our expectation that inflammatory responses (e.g. APRs) relate to the local and current conditions experienced by birds, because they are mounted over very short periods of time (minutes to hours). We therefore conclude that the APR for skylarks experiencing the annual environmental variation in the Netherlands is similar in magnitude in all seasons, and crucial enough to be not traded off against other annual cycle functions.

### Consistent induced responses but variable baseline levels

The lack of seasonal modulation in the magnitude of an APR contrasts with the literature and with the results of a related study in which we measured constitutive immunological parameters in unchallenged skylarks. In this study, lysis and agglutination titers, haptoglobin concentrations, and proportions of eosinophils, basophils and monocytes differed among annual cycle stages when measured directly upon capture in the field [41]. Studies on other species also find reductions in particular immunological components during specific annual cycle stages [3], [8]–[12], [62], [63]. Taken together, these studies point out that seasonal modulations of immune systems differ among species, environments, life-histories, and, importantly, immune parameters. Our skylark results develop this further by showing that within a species baseline values of some immune indices were seasonally variable, but the magnitude of response to a standard inflammatory challenge was seasonally consistent. Overall, these studies identify some interpretational limitations of different approaches as well as the importance of distinguishing between baseline values and induced responses when studying ecological immunology. Furthermore, the contrasting results, in effect, make the case for measuring both baselines and response when linking ecology, evolution and immunology.

### Physiological responses to LPS injection

Significant changes in six of the seven measured parameters indicated that the LPS injection successfully triggered an inflammatory response. The increase in nightly metabolic rate, BMR and body temperature fall within the expected costs of fever [64]. The average increase in metabolic rate (regardless of stage) was similar to the increases reported in other studies after immune challenges in single annual cycle stages [4], [12], [65], [66]. Thus, inflammation-associated metabolic costs may be fairly conserved among avian species and not simply consistent among annual cycle stages within skylarks.

LPS-injected skylarks lost on average 13.6% more body mass over night than control birds. It has been suggested that mass losses following an LPS injection are mainly due to sickness-related anorexia [16], [19] rather than metabolic costs per se. As we measured mass loss over the night while all birds were resting and none had access to food, our estimates of mass loss reflect a true metabolic cost. Likewise, experimental birds had significantly higher ketone concentrations, which reflect lipid catabolism during fasting [37], and this elevation can be seen as a direct consequence of the LPS-induced metabolic changes. Body mass at the point of capture in the field did not predict mass loss over the metabolic measurement period, even though body mass showed strong seasonal variation. Thus, our data do not support the idea that energy stores are a proximate mechanism for seasonal modulations in immune defences [16], [19].

Although we found sex differences in most parameters, we found no evidence that the LPS injection had different effects in males and females. We acknowledge that sample sizes per sex are limited and that a lack of sex*treatment interactions could be due to low statistical power. However, Owen-Ashley et al. [20] also find little evidence for sex differences in LPS-induced sickness behaviour in white-crowned sparrows *Zonotrichia leucophrys*. These results contrast with the idea that the two sexes allocate resources to the immune system differently [2], [14] but support our hypothesis that the APR is critically important and always maintained.

### Conclusions

We found no evidence for seasonal modulation of acute phase responses among the five distinct annual cycle stages of a wild temperate zone bird, even though energy budgets show strong seasonal variation. Skylarks undergoing an experimentally triggered inflammatory response exhibited increases in metabolic rate, body mass loss, body temperature and ketone concentration, and these changes demonstrate energetic costs of an APR. The consistent lack of interaction between treatment and annual cycle stage suggests that the acute phase response is an essential immunological defence, one that is too crucial for survival to be compromised through trade-offs with other life-history activities despite the response's clear costs.
